# Early Shear Failure of a 3-Peg Modified Dome Patellar Implant

**DOI:** 10.1016/j.artd.2024.101448

**Published:** 2024-07-04

**Authors:** Yifan V. Mao, Matthew V. Dipane, Erik N. Zeegen, Edward J. McPherson

**Affiliations:** a UCLA David Geffen School of Medicine, Los Angeles, CA, USA; b Department of Orthopaedic Surgery, UCLA Health, Santa Monica, CA, USA

**Keywords:** Patella, Mechanical failure, All-polyethylene, Modified dome, Mechanical shear, Total knee arthroplasty

## Abstract

Patellar failure in total knee arthroplasty is a major source of complications postoperatively. Previous patellar failure reports commonly cited dissociations of modular and metal-backed patellar implants. However, mechanical breakage of monoblock all-polyethylene patellar implants is very rare. We present a case of an early shear failure of a 3-peg modified dome all-polyethylene patellar implant at 16 months. The patient underwent a revision procedure and at 1-year follow-up, the patient’s patella and knee remained stable with no reported issues. Shear failure of polyethylene pegs requires excess cyclic shear stress imparted at the prosthetic-bone interface. Patellar implants with a cone design are more constrained and, if misaligned relative to the metallic trochlea, may impart excess shear force to the patella during flexion.

## Introduction

Complications involving the patellofemoral articulation in primary total knee arthroplasty (TKA) are not infrequent [[1], [2], [3]], but mechanical breakage of patellar implants is rare. Implant breakage is usually associated with dissociation of modular patellar implants [4,5]. More unusual is the mechanical breakage of monoblock polyethylene implants. This report describes the early breakage of a modern modified dome all-polyethylene patellar implant whereby the implant sheared off from its 3 pegs. We review mechanisms that may have contributed to this observed failure.

## Case history

### Demographics

**Figure 1:**
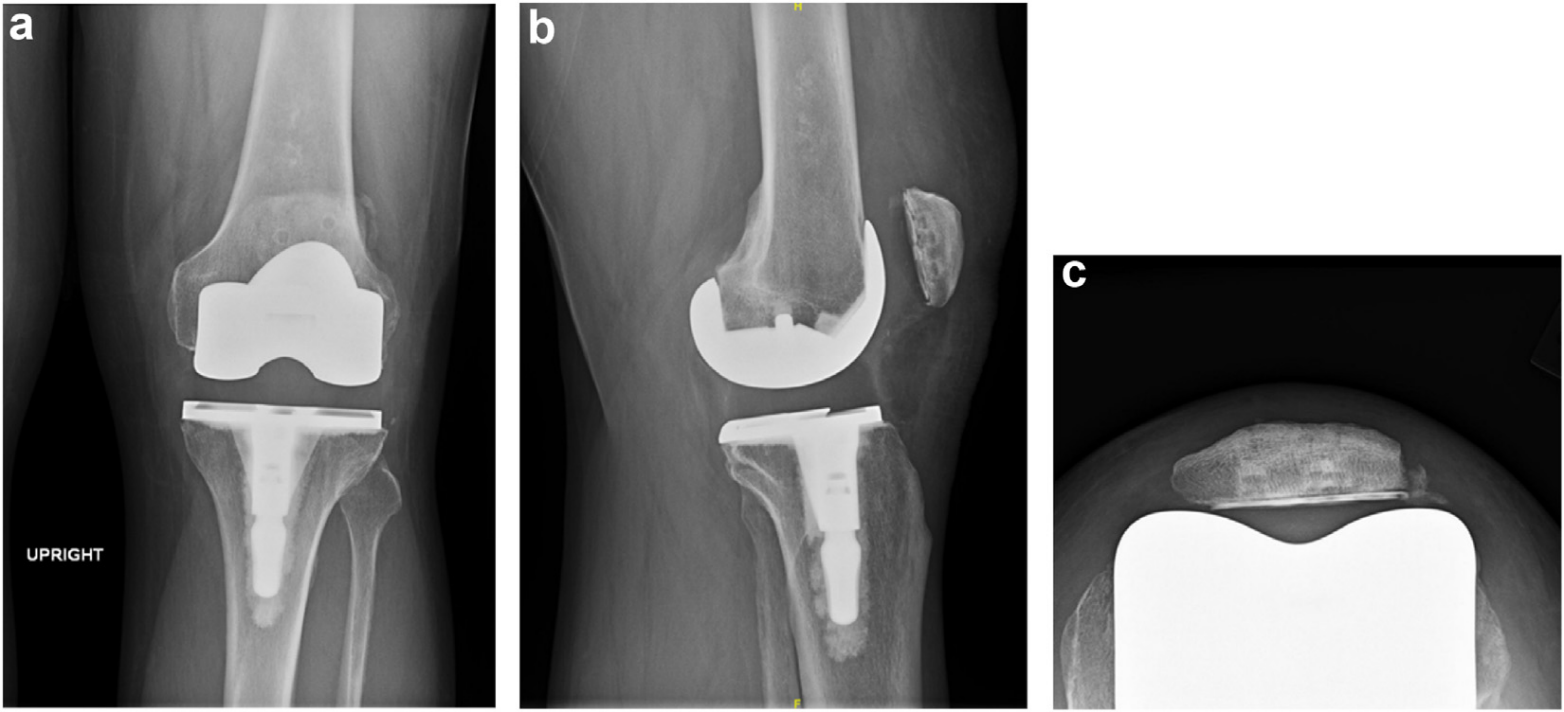
(a-c) Prefailure radiographs at 4 months postoperatively of primary TKA showing anteroposterior, lateral, and sunrise views. Note the radiolucency between the cement and the patellar bone, best observed on the sunrise view. In hindsight, this may have been an early indication of failure at the cement-bone interface.

A 52-year-old man with end-stage gonarthrosis underwent a left TKA in November 2021. The preoperative knee range of motion was 10 to 115 degrees. Standing radiographic hip-knee-ankle angle measured 178 degrees (net valgus attitude) with Kellgren-Lawrence 4 degeneration of the medial compartment. Prefailure radiographs at 4 months postoperatively of primary TKA are shown in Figure 1a-c. His medical history was relevant for osteoarthritis and a body mass index of 37 kg/m^2^. He enjoyed light recreational activities on a weekly basis.

### Procedure

The primary TKA was performed using a medial parapatellar arthrotomy. The knee implant used was the Persona Knee System (Zimmer-Biomet, Warsaw, IN). A posterior stabilized femur was utilized, mated with a metal base plate and constrained posterior stabilized vitamin E reinforced bearing. Bone cuts were made with the Robotic Surgical Assistant (ROSA, Zimmer-Biomet, Montreal, CA). The selected limb alignment was 2 degrees varus from his mechanical axis. Knee balance and femoral implant rotational bone cuts were performed using a gap balance technique. The patella was prepared with a manual saw cut, first measuring the patellar height with a caliper. The patellar implant used was 41mm modified dome vitamin E reinforced all-polyethylene with 3 pegs. The patella, tibia, and femur were cemented with the same batch of Simplex P cement (Howmedica Osteonics Corp, Mahwa, NJ). Two bags of low-viscosity cement with 1 gram of vancomycin per bag was mixed. All bone surfaces were prepared with pulsed saline mechanical lavage. Cement was placed only on the patellar bony surface. The patellar button was placed by hand into position with all 3 pegs into the holes, then compressed with the patellar clamp. While the cement was curing, the knee was held in full extension with axial loading and with a trial polyethylene liner in place. The patella tracked centrally at the time of closure testing by the “no thumb” technique after tourniquet deflation [6]. A lateral retinacular release was not performed. The intraoperative knee range measured 0 to 120 degrees. He underwent an uneventful recovery, and his right knee was similarly replaced 11 months later, recovering without event.

### Clinical follow-up

**Figure 2:**
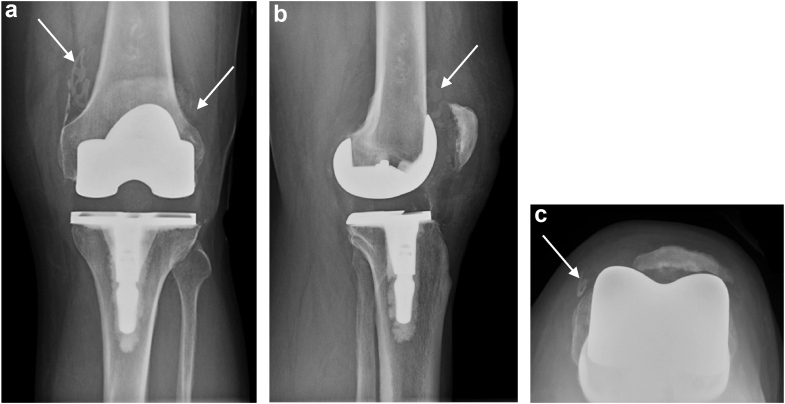
(a-c) Postfailure radiographs at 16 months postoperatively of primary TKA with failed patella implant. Anteroposterior radiograph (a) shows well-fixed implants, soft-tissue swelling, and a knee effusion. Arrows point to displaced polyethylene patella fragments. In lateral view (b), the patellar implant shadow is seen within the supra-patellar pouch (arrow). (c) shows sunrise view with absent polyethylene implant shadow. Note peg cement shadows that suggest the polyethylene pegs are still within the cement. Arrow points to a patella fragment.

He returned at the 16-month follow-up, complaining of left knee pain and swelling. He reported no falls nor antecedent trauma. One month prior, he began using an elliptical machine and riding a stationary bicycle for conditioning. Prior to this, his reported activity level was typical activities of daily living with an occasional 1-mile walk for exercise. The knee was warm with a moderate effusion, but no erythema or abscesses were observed. The knee was stable through its range of 0 to 120 degrees, but with patellar crepitus. Standing radiographic hip-knee-ankle angle measured 180 degrees (net neutral alignment). Knee radiographs (Fig. 2a-c) showed a free-floating polyethylene implant within the suprapatellar pouch. The polyethylene pegs appeared to remain fixed within the patellar bone, suggesting shear failure of the all-polyethylene implant. Arthrocentesis showed serous, blood-tinged fluid with a negative string sign. Synovial fluid markers for infection were negative including Next Generation Microbial DNA sequencing (MicroGenDX, Lubbock, TX), culture, alpha defensin, and synovial c-reactive protein (CD Laboratories, Baltimore, MD). The synovial white blood cell count was 100 cells/μL with 22% segmented neutrophils.

### Revision procedure

**Figure 3:**
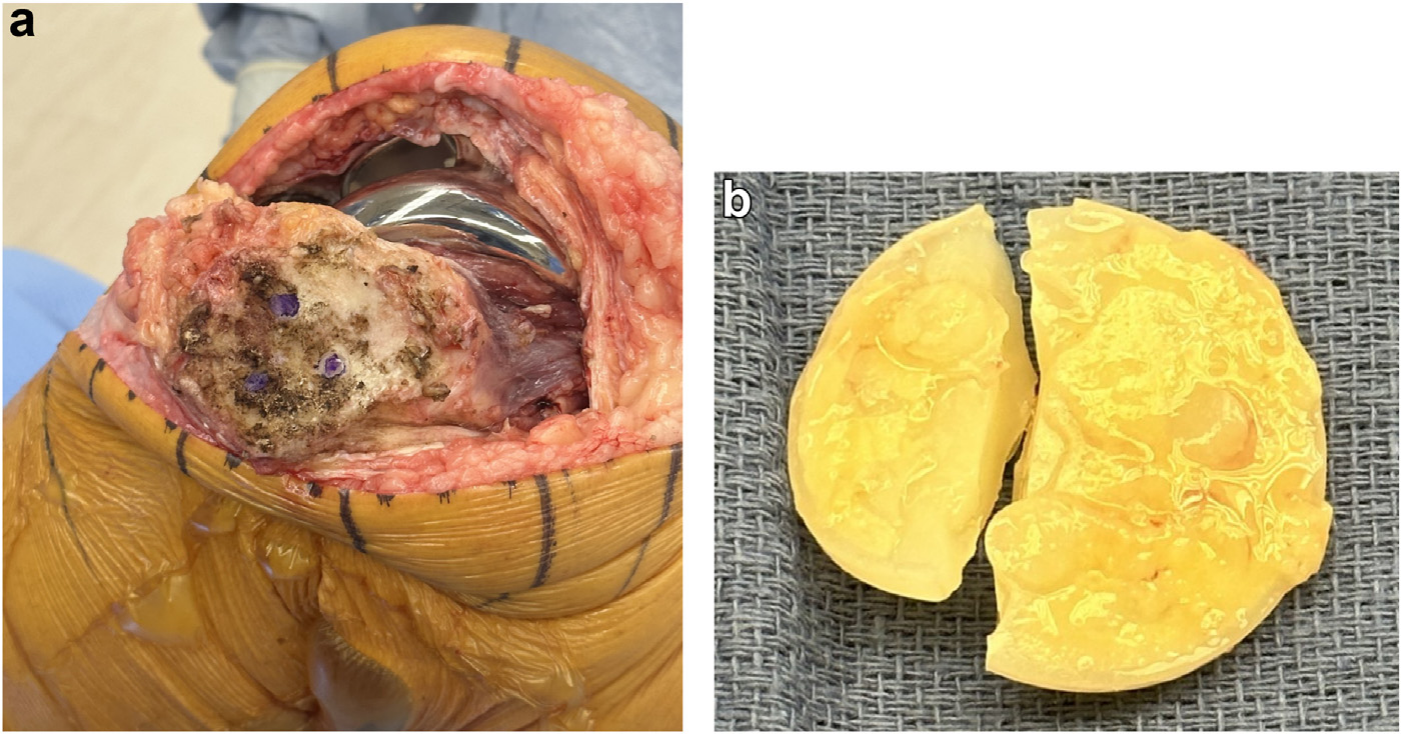
(a and b) Intraoperative findings of failed patella implant. (a) shows the debrided patella with the 3 polyethylene pegs well-fixed within the patella (marked with violet pen). (b) shows the fractured polyethylene button, which was located in the medial gutter.

**Figure 4:**
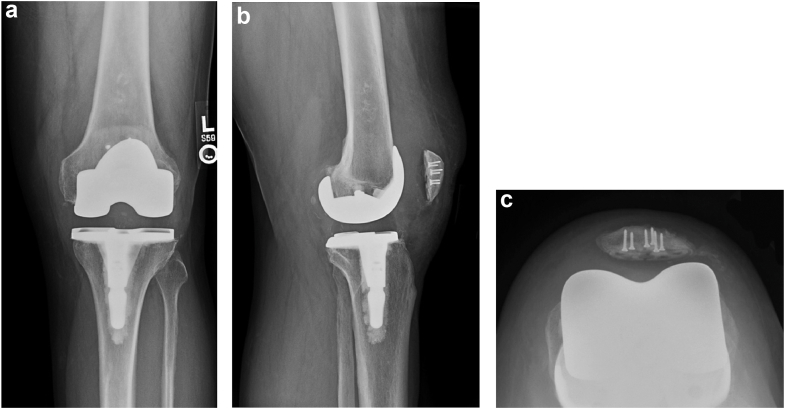
(a-c) Radiographs of revised patella 12 months postoperative. Anteroposterior radiograph (a) shows patella centrally overlying the trochlea. Lateral view (b) of patella demonstrates the cement-bone interface augmented with patellar rebar screws placed into the dorsal cortex. The sunrise view (c) suggests subtle residual lateral patellar tilt.

In May 2023, a revision procedure was performed consisting of an extended arthrotomy, synovectomy, modular tibial bearing exchange, and revision of the patellar implant. Figure 3a shows the intraoperative findings of the patellar implant’s shearing from its 3 pegs, which were still well fixed within bone. In addition, the patellar implant had broken into 2 pieces (Fig. 3b). The patellar pegs were removed with the Ultra-Drive (Zimmer-Biomet), an ultrasonic cement removal device, and the patellar bone deficiencies were augmented with 6 2.0-m patellar rebar screws placed vertically [7]. Another patellar implant of the same design and size was cemented into the patella using 3 new peg holes drilled adjacent to the prior holes. After performing a lateral retinacular release, the revised patella showed central patellar tracking testing with a “no thumbs” technique. Radiographs of the reconstructed patella are shown in Figure 4a-c. He underwent an uneventful recovery and, at 12 months postoperatively, reports returning to all customary activities with good pain relief.

## Discussion

Mechanical breakage of all-polyethylene patellar implants is rare. In our literature review, we identified only 6 cases of isolated mechanical all-polyethylene patella breakage [4,5,8,9]. Stulberg et al. [10] reported a case of bilateral patellar component failure due to fatigue fracture of all-polyethylene fixation pegs in a highly cross-linked ultra-high-molecular weight polyethylene design. Among 953 cases with all-polyethylene patellar components, Huang et al. [4] reported 4 cases of breakage of the patellar component at the peg-button interfaces. Francke and Lachiewicz [5] report one case of shear failure of all 3 fixation pegs and loosening of the component in a cemented all-polyethylene patellar component. Proposed contributors of failure included patient related factors of obesity, high activity level, and patellar osteonecrosis [4,5,8,9]. Other reported factors included patellar mal-alignment, inadequate cement fixation (to bone and implant), and mechanical peg weakness [4,5,8,9]. Unique to this present case is failure early in the life cycle of the implant.

Our explanation for the early failure is mechanical shear. Intraoperative inspection showed all 3 pegs solidly fixed with cement in the patellar bone, which required removal with ultrasonic tools. All pegs failed at the level of the prosthetic-bone interface, similar to the failures reported by Stulberg and by Huang [4,10]. We believe multiple causative factors were involved in this case, resulting in a “perfect storm” creating shear forces great enough to cause early failure. In Table 1, we summarize factors that may contribute to increased patellar shear.

**Table 1:** Factors affecting shear stress at patella-bone interface.

Factor	Desired	Detrimental
Trochlear/Patellar Shape	Unconstrained	Constrained
Coronal misalignment	Low Q angle	High Q angle
Transverse misalignment	External rotation femur	Internal rotation femur
Peg number	3-peg	1-peg
Peg distance to center	Greater distance	Shorter distance
Peg diameter	Large diameter	Small diameter
Backside cement inlay area	High surface area	Low surface area
Cement	High mechanical strength	Reduced mechanical strength

**Figure 5:**
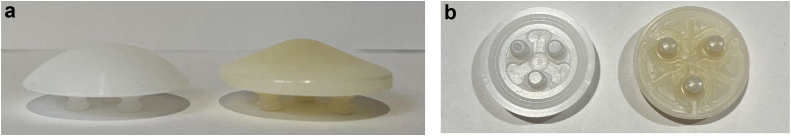
(a and b) Images comparing Vanguard dome patella (left) to Persona cone patella (right) with implants of similar size. (a) shows the side view and (b) shows the backside view. A comparison of features is listed in Table 2.

One factor we believe pertinent is the trochlear/patellar implant shape. The Persona Modified Dome patella closely resembles a cone design (Fig. 5a) [11], as opposed to the more common standard dome design. This cone design confers more constraint, as during flexion, the patella enters into the cone shaped metallic trochlear groove and congruently mates with the trochlea. If, however, the extensor mechanism is misaligned with the trochlea, the prosthetic trochlea groove will force the patella into the central channel. Consequently, a mechanical shear force is created, as seen in this case. We theorize that there was an extensor mating mismatch significant enough to cyclically fatigue the polyethylene pegs. To our knowledge, this is the first reported peg failure of a patella with a cone-like design.

We suspect the second important factor at play was implant misalignment, as previously cited by Shafi [12]. Misalignment can occur both in the coronal and transverse planes. In the coronal plane, an excess Q angle increases lateral subluxation forces, imparting increased shear forces to a constrained patellar implant. In this case, the radiographic hip-knee-ankle angle was 180 degrees, making this a noncontributing factor. However, transverse misalignment of the femoral component may have been a contributing factor. Tapasvi et al. [9], in a randomized study comparing gap balancing vs measured resection technique in primary TKA, demonstrated increased rotational variability of femoral implants with the gap balancing technique. In our case, this may have created a subtle misalignment with an internally rotated femur. In retrospect, a CT scan evaluating femoral component rotation prior to revision would have been helpful.

Finally, patellar design may have contributed to this observed failure. First, large-diameter pegs are more robust and less likely to cyclically deform with shear and bending stress. Second, multiple pegs seated within bone provides increased surface contact with bone and confers increased resistance against shear. Third, in 3-peg designs, pegs located farther from the patellar center are typically better able to resist deformational forces, including shear. Finally, the polyethylene material itself could be a contributing factor by fact of having reduced mechanical properties. It is known that highly irradiated crosslinked polyethylene produced via a heat anneal process has reduced mechanical properties compared to the same polyethylene having not undergone the crosslinking process [13]. Figure 5a and b and Table 2 compare the design features of the Persona Modified Dome patella to the Vanguard (Zimmer Biomet, Warsaw, IN.) low profile dome design, an implant with no reported mechanical breakage. In comparison, the only major notable differences between these 2 frequently used designs are the shape of the patella and the type of polyethylene material used. These 2 differences may serve as a starting point in studying patellar shear failure in all-polyethylene patellar implants.

**Table 2:** Comparison of persona modified dome cone-shaped 3-peg patella and vanguard low profile dome 3-peg patella.

Design measurements	Persona modified dome cone-shaped 3-peg patella	Vanguard low profile dome 3-peg patella
Implant diameter	38.0 mm	37.0 mm
Total implant height	9.4 mm	8.4 mm
Peg height	5.3 mm	4.7 mm
Peg distance to center	8.3 mm	6.0 mm
Peg minimum diameter	3.7 mm	3.7 mm
Peg maximum diameter	5.9 mm	5.8 mm
Polyethylene processing	Highly crosslinked irradiated, heat annealed with vitamin E, machined	Compression molded, machined

This report has limitations. This is the report of a single case, which confers limited generalizability and no ability to establish cause-effect relationships. Furthermore, we did not perform mechanical testing to validate our proposed mechanisms of failure. However, the report highlights avenues for potential future testing to create a shear model for patella implant failure in all-polyethylene implants.

## Summary

Before the present report, to our knowledge, no mechanical failure of an all-polyethylene “modified dome” patellar component had been reported. Shear failure of polyethylene pegs requires excess cyclic shear stress imparted at the prosthetic-bone interface. Cone-shaped patellar implants are more constrained, and if misaligned relative to the metallic trochlea, may impart excess shear forces to the patella. Even though the early postoperative radiographs showed good cement technique and the patella tracked normally, a combination of factors including patella implant design, material factors, and gap balancing surgical technique may have contributed to the early shear failure of this patellar component.

## Conflicts of interest

Erik Zeegen reports being a paid consultant for Zimmer-Biomet, J&J/Ethicon, having stock in RadLink Inc.; received research support from Zimmer-Biomet; being a part of editorial board for Journal of Arthroplasty, Arthroplasty Today, Arthroplasty; and being a a board member for American Association of Hip and Knee Surgeons. Edward McPherson reports receiving royalties from Zimmer-Biomet; being a part of speakers bureau for Austin Medical Ventures, Zimmer-Biomet; being a paid consultant for Austin Medical Ventures, Zimmer-Biomet; being a part of editorial board for Reconstructive Review. All other authors declare no potential conflicts of interest.

For full disclosure statements refer to https://doi.org/10.1016/j.artd.2024.101448.

## Informed patient consent

The author(s) confirm that written informed consent has been obtained from the involved patient(s) or if appropriate from the parent, guardian, power of attorney of the involved patient(s); and, they have given approval for this information to be published in this case report (series).

## CRediT authorship contribution statement

**Yifan V. Mao:** Writing – review & editing, Writing – original draft. **Matthew V. Dipane:** Writing – review & editing, Project administration. **Erik N. Zeegen:** Writing - review & editing, Investigation, Supervision, Data curation, Conceptualization. **Edward J. McPherson:** Writing – review & editing, Writing – original draft, Supervision, Investigation, Conceptualization.

## Appendix A Supplementary data

Conflict of Interest Statement for Dipane

Conflict of Interest Statement for McPherson

Conflict of Interest Statement for Zeegen

Conflict of Interest Statement for Mao
