# Supplementary material for: Early Shear Failure of a 3-Peg Modified Dome Patellar Implant
Source: Arthroplast Today. 2024 Jul 4;28:101448. doi: 10.1016/j.artd.2024.101448 (PMC11283063; doi:10.1016/j.artd.2024.101448)
Supplement: Conflict of Interest Statement for Zeegen [file mmc3.pdf]

INDIVIDUAL CONFLICT OF INTEREST STATEMENT

American Association of Hip and Knee Surgeons

(Adopted from the American Academy of Orthopaedic Surgeons disclosure statement)

The following form **must be filled out completely and submitted by each author (example, 6 authors, 6 forms). All items require a response. If there is no relevant disclosure for a given item, enter "*None.*"**

Manuscript Title: Early Shear Failure of a Three-Peg V-Design Patellar Implant: A Case Report

1. Royalties from a company or supplier (The following conflicts were disclosed)

   None.

2. Speakers bureau/paid presentations for a company or supplier (The following conflicts were disclosed)

   None.

3A. Paid employee for a company or supplier (The following conflicts were disclosed)

   None.

3B. Paid consultant for a company or supplier (The following conflicts were disclosed)

   Zimmer-Biomet, J&J/Ethicon.

3C. Unpaid consultants for a company or supplier (The following conflicts were disclosed)

   None.

4. Stock or stock options in a company or supplier (The following conflicts were disclosed)

   RadLink Inc.

5. Research support from a company or supplier as a Principal Investigator (The following conflicts were disclosed)

   Zimmer-Biomet.

6. Other financial or material support from a company or supplier (The following conflicts were disclosed)

   None.

7. Royalties, financial or material support from publishers (The following conflicts were disclosed)

   None.

8. Medical/Orthopaedic publications editorial/governing board (The following conflicts were disclosed)

   Journal of Arthroplasty, Arthroplasty Today, Arthroplasty.

9. Board member/committee appointments for a society (The following conflicts were disclosed)

   American Association of Hip and Knee Surgeons

Each author must sign AND print or type his/her name, date and submit a separate form

In addition, one BLINDED Conflict of Interest form (no author names used) should be submitted per manuscript with all author disclosures.

Erik N. Zeegen, MD	[signature]	4/1/24
Author Name (Print or Type)	Author Signature	Date
